# Time trends in breast cancer incidence and mortality in a mid-sized northeastern Brazilian city

**DOI:** 10.1186/1471-2458-12-883

**Published:** 2012-10-19

**Authors:** Carlos Anselmo Lima, Margareth Rose Uchoa Rangel, Matheus Macedo-Lima, Angela Maria da Silva

**Affiliations:** 1 Núcleo de Pós-graduação em Medicina da Universidade Federal de Sergipe, Rua Claudio Batista s/n B Santo Antonio, Aracaju, SE, 49060-100, Brazil; 2 Registro de Câncer de Base Populacional de Aracaju, Av Tancredo Neves s/n B. Capucho, Aracaju, SE, 49095-000, Brazil; 3 Hospital Universitário/Universidade Federal de Sergipe, Rua Claudio Batista s/n B Santo Antonio, Aracaju, SE, 49060-100, Brazil; 4 Universidade Federal de Sergipe, Av Marechal Rondon s/n, São Cristovão, SE, 49100-000, Brazil

**Keywords:** Breast cancer, Incidence, Mortality, Cancer registry, Time trends

## Abstract

**Background:**

Breast cancer incidence within an area is usually proportional to the area’s income level. High-income areas have shown the highest incidence rates and since 2003, negative trends. As for mortality, rates are often higher in low-income regions. The purpose of this study was to analyze trends in incidence and mortality in a capital city of a northeastern Brazilian state with an intermediate human development index.

**Methods:**

Incidence data from the Population-Based Cancer Registry of Aracaju and mortality data from the Official State Database for the period 1996–2006 were used. Incidence and mortality crude and age-standardized rates were calculated. Time trends were obtained using the Joinpoint Regression Model.

**Results:**

For the period studied, invasive breast cancer age-standardized incidence rates increased annually with an annual percentage change (APC) of 2.9 (95% CI: 1.2-4.6). Significant increasing trends were observed in groups aged 45–54 years (APC: 3.9, 95% CI: 1.4 to 6.6), and 55–64 years (APC: 5.6, 95% CI: 1.8 to 9.6). Age-standardized mortality rates did not show an increasing trend (APC: 3.0, (95% CI: -2.8 to9.1), except for the group aged 55–64 years (APC: 11.3, 95% CI: 1.1 to 22.4).

**Conclusions:**

In the study community, breast cancer showed increasing incidence among women in the peri- and postmenopausal periods. However, mortality did not present increasing overall trends, except for among the group aged 55–64 years. For better outcomes, screening policies should focus on the peri- and postmenopausal periods of women’s lives to diagnose disease.

## Background

In 2008, breast cancer was the most common female neoplasm in both low- and high-income countries, and accounted for 23% of all incident cases and 14% of all female deaths worldwide. It presents a great burden on women because it is the leading cause of cancer related death among them worldwide [1,2]. In Brazil, cancer incidence estimates for 2012 include a crude rate for breast cancer of 52.50/100,000. For the State of Sergipe, the crude rate has been estimated at 34.95/100,000 and for the capital Aracaju, at 57.76/100,000 [3].

The highest incidences of breast cancer are in high-income countries of North America, Europe and Australasia. High incidence also occurs in low- and middle-income areas, where low fat ingestion has been observed, calling attention to the multifactorial nature of breast cancer development [4,5]. Conversely, for breast cancer mortality, higher rates have been observed in lower income areas, probably reflecting late diagnosis and inadequate access to treatment facilities [6].

Disparities in breast cancer incidence and mortality have been well discussed and linked to several causes. Screening mammography, which has been performed since the 1980s, has played an important role in early cancer diagnosis. While screening has led to trends of decreasing mortality, it has only done so in places where screening has been systematic and the population has had access to good quality mammography [7]. Decreasing incidence trends have been reported in high-income countries since 2003, following the Women’s Health Initiative report that led to a dramatic decline in hormone replacement therapy (HRT) use [8,9]. However, the impact on mortality needs to be verified.

It is the aim of the present study to analyze time trends in breast cancer incidence and mortality in a mid-income town of northeastern Brazil and offer means to implement control policies to reduce mortality from this common cancer.

## Methods

Aracaju is the capital of the State of Sergipe, northeastern Brazil, and is located at 10°54`40``S 37°04`18``W, has a population of 571,149, predominantly urban, and a Human Development Index (HDI) of 0.794 [10].

This was an ecological study of time trends that aimed to describe changes in breast cancer incidence and mortality in Aracaju. Data of all invasive breast cancer cases were obtained from the Cancer Registry of Aracaju, which has collected incident case data since 1996. A database excluding in situ and non-melanoma skin cancer was specially prepared for the study. The Cancer Registry of Aracaju has actively collected cancer case data from public and private sources, especially medical facilities that provide cancer treatment. Some government databases such as the information systems on hospital and outpatient procedures, the information systems on breast and cervical cancer, and the mortality system were also used to identify cases and complete information of incident cases. Mortality data were retrieved from the Mortality Database of the State of Sergipe, which provided information for the Brazilian Mortality Database. Mid-year female population data by year for the Municipality of Aracaju were obtained from the official source, DATASUS. Initially, five-year age groups were created, then age groups (<45, 45–54, 55–64, and 65+ years) were created to approximate premenopause, perimenopause, the first ten years of the postmenopausal period, and thereafter, respectively.

The Cancer Registry of Aracaju has been officially integrated to the Brazilian National Cancer Institute (INCA) and has followed the rules determined by the International Agency for Research on Cancer (IARC). For the period 1996 to 2006, all cases of invasive breast cancer were included in the registry. Crude rates (CRs) and age-standardized rates (ASRs), adjusted by the World Population [11,12], were calculated using Cancer Registry software [13]. Ninety-five percent confidence intervals (95% CI) were calculated by the formula 95% CI = R+/− (1.96xSE), where R was the annual rate and SE the standard error. The standard error was calculated by the formula SE = R/√N, where R was the annual rate and N the number of cases per year. The mortality/incidence ratio was calculated using CRs to provide an indication of the prognosis of breast cancer patients. The Joinpoint Regression Program [14] was used to calculate time trends in incidence and mortality with a model based on the assumption of a minimal number of join points where statistically significant changes in time trends occur. It was a logarithmic linear model that added join points, from 0 to 5, and calculated the difference up to a statistically significant value, using the Monte Carlo Permutation Test [15]. Thus, the Annual Percent Change (APC) was calculated, and time trends in breast cancer incidence and mortality were defined. Time trends for the consecutive eleven-year series were calculated using CRs, ASRs, and age-specific rates (age groups <45, 45–54, 55–64, and 65+ years) as the dependent variables; the year as the independent variable and the Input Standard Error of Dependent Variable was chosen with appropriate standard error calculations (formula mentioned above).

## Results

During the study period, 1,264 cases of invasive breast cancer and 336 breast cancer deaths were identified. Age-specific, crude and age-standardized breast cancer incidence (Table 1) and mortality (Table 2) rates are presented. Some fluctuation was observed in the annual rates, but no special reason could be found for it. Crude incidence rates varied from 38.2/100,000 (95% CI: 30.2 to 46.2) and 32.9/100,000 (95% CI: 25.5 to 40.2) in the early years (1996 and 1997) to 54.3/100,000 (95% CI: 45.4 to 63.1) and 52.4/100,000 (95% CI: 43.8 to 61.1) in the later years of the series (2005 and 2006). Compared with the <45 age group, higher rates were observed in the perimenopausal period, and rates were highest in the postmenopausal periods of women’s lives. Age-standardized incidence rates varied from 49.6/100,000 (95% CI: 39.2 to 60.0) and 42.5/100,000 (95% CI: 33.0 to 52.1) in the early years to 64.0/100,000 (95% CI: 53.5 to 74.4) and 60.8/100,000 (95% CI: 50.8 to 70.8) in the later years of the series. Mortality rates also fluctuated over the years. CRs varied from 9.2/100,000 (95% CI: 5.2 to 13.3) in 1996 and 8.6/100,000 (95% CI: 4.9 to 12.4) in 1997 to 16.2/100,000 (95% CI: 11.4 to 21.0) in 2005 and 12.6/100,000 (95% CI: 8.4 to 16.9) in 2006. Age-specific mortality rates were higher in the postmenopausal period. Age-standardized mortality rates were 11.4/100,000 (95% CI: 6.5 to 16.2) in 1996, climbed to 21.4/100,000 (95% CI: 15.3 to 27.6) in 2001, then dropped to 14.7/100,000 (95% CI: 9.8 to 19.6) in 2006. The mean mortality-to-incidence ratio for the time series was 0.27.

**Table 1:** Breast cancer incidence, with age-specific case counts (N), crude rates (CR) and age-standardized rates (ASR), Aracaju, Sergipe, Brazil, 1996–2006

**Year**	**Age**	**N**	**CR (95% CI)**	**ASR (95% CI)**
1996	All	87	38.2 (30.2-46.2)	49.6 (39.2-60.0)
	<45	21	26.2 (15.0-37.4)	
	45–54	21	134.8 (77.1-192.4)	
	55–64	17	150.5 (78.9-222.0)	
	65+	28	258.4 (162.7-354.1)	
1997	All	76	32.9 (25.5-40.2)	42.5 (33.0-52.1)
	<45	23	29.6 (17.5-41.7)	
	45–54	20	103.1 (57.9-148.4)	
	55–64	11	98.4 (40.3-156.6)	
	65+	22	156.3 (91.0-221.6)	
1998	All	90	38.4 (30.5-46.4)	50.5 (40.1-61.0)
	<45	19	23.3 (12.8-33.8)	
	45–54	23	150.1 (88.7-211.3)	
	55–64	20	178.0 (100.0-256.0)	
	65+	28	270.5 (170.3-370.6)	
1999	All	104	43.9 (35.4-52.3)	55.8 (45.1-66.5)
	<45	24	29.5 (17.7-41.3)	
	45–54	28	162.0 (102.0-221.9)	
	55–64	20	177.5 (99.7-255.3)	
	65+	32	307.4 (200.9-413.9)	
2000	All	115	46.8 (38.3-55.4)	53.6 (43.8-63.4)
	<45	29	33.3 (21.2-45.4)	
	45–54	40	156.8 (108.2-205.3)	
	55–64	17	133.0 (69.8-196.3)	
	65+	28	211.4 (133.1-289.7)	
	Unknown	1		
2001	All	118	47.3 (38.8-55.9)	56.5 (46.3-66.7)
	<45	24	25.7 (15.4-35.9)	
	45–54	26	175.2 (107.9-242.6)	
	55–64	30	231.9 (148.9-314.9)	
	65+	37	250.8 (170.0-331.7)	
	Unknown	1		
2002	All	141	55.9 (46.7-65.1)	63.5 (53.0-74.0)
	<45	38	42.1 (28.7-55.5)	
	45–54	47	186.2 (133.0-239.5)	
	55–64	23	174.5 (103.2-245.8)	
	65+	32	230.8 (150.8-310.8)	
	Unknown	1		
2003	All	112	43.9 (35.7-52.0)	52.0 (42.4-61.7)
	<45	26	30.4 (18.7-42.1)	
	45–54	29	152.8 (97.2-208.5)	
	55–64	26	181.9 (112.0-251.8)	
	65+	31	199.9 (129.5-270.3)	
2004	All	136	52.6 (43.8-61.5)	61.5 (51.2-71.8)
	<45	32	34.5 (22.5-46.5)	
	45–54	31	181.8 (117.8-245.8)	
	55–64	32	232.4 (151.9-312.9)	
	65+	41	291.2 (202.0-380-3)	
2005	All	144	54.3 (45.4-63.1)	64.0 (53.5-74.4)
	<45	26	28.3 (17.4-39.2)	
	45–54	35	181.0 (121.0-241.0)	
	55–64	32	219.2 (143.2-295.1)	
	65+	51	363.5 (263.7-463.3)	
2006	All	141	52.4 (43.8-61.1)	60.8 (50.8-70.8)
	<45	29	30.4 (19.3-41.4)	
	45–54	32	188.0 (122.9-253.1)	
	55–64	35	243.7 (162.9-324.4)	
	65+	44	337.4 (237.7-437.1)	
	Unknown	1		

**Table 2:** Breast cancer mortality, with age-specific case counts (N), crude rates (CR) and age-standardized rates (ASR), Aracaju, Sergipe, Brazil, 1996–2006

**Year**	**Age**	**N**	**CR (95% CI)**	**ASR (95% CI)**
1996	All	20	9.2 (5.2-13.3)	11.4 (6.5-16.2)
	<45	8	9.9 (3.0-16.7)	
	45–54	4	25.5 (0.5-50.4)	
	55–64	2	18.6 (−7.2-44.5)	
	65+	6	57.7 (11.5-103.9)	
1997	All	20	8.6 (4.9-12.4)	11.4 (6.4-16.4)
	<45	4	5.0 (0.1-9.9)	
	45–54	1	6.3 (−6.0-18.6)	
	55–64	4	35.0 (0.7-69.3)	
	65+	11	108.5 (44.4-172.6)	
1998	All	24	10.2 (6.1-14.3)	13.6 (8.1-19.0)
	<45	4	5.0 (0.1-10.0)	
	45–54	7	38.6 (10.0-67.2)	
	55–64	5	48.0 (5.9-90.1)	
	65+	8	96.2 (29.5-162.9)	
1999	All	28	11.8 (7.4-16.2)	14.5 (9.1-19.8)
	<45	6	7.8 (1.6-14.1)	
	45–54	10	51.8 (19.7-84.0)	
	55–64	2	17.9 (−6.9-42.8)	
	65+	10	106.5 (40.5-172.5)	
2000	All	31	12.6 (8.2-17.1)	13.8 (9.0-18.7)
	<45	10	11.3 (4.3-18.3)	
	45–54	12	52.4 (22.7-82.0)	
	55–64	3	20.7 (−2.7-44.2)	
	65+	6	46.2 (9.2-83.2)	
	Unknown			
2001	All	47	18.9 (13.5-24.2)	21.4 (15.3-27.6)
	<45	7	7.3 (1.9-12.8)	
	45–54	17	78.5 (41.2-115.8)	
	55–64	8	60.6 (18.6-102.6)	
	65+	15	135.0 (66.7-203.4)	
	Unknown			
2002	All	23	9.1 (5.4-12.8)	10.2 (6.0-14.3)
	<45	5	5.8 (0.7-10.9)	
	45–54	7	29.8 (7.7-51.9)	
	55–64	4	31.5 (0.6-62.3)	
	65+	7	67.0 (17.4-116.7)	
	Unknown			
2003	All	41	16.1 (11.1-21.0)	20.1 (13.9-26.2)
	<45	7	7.5 (1.9-13.0)	
	45–54	9	40.2 (13.9-66.5)	
	55–64	13	98.4 (44.9-151.9)	
	65+	12	83.9 (36.4-131.4)	
2004	All	25	9.7 (5.9-13.5)	11.5 (7.0-15.9)
	<45	5	5.4 (0.7-10.2)	
	45–54	2	8.3 (−3.2-19.9)	
	55–64	9	62.1 (21.5-102.7)	
	65+	9	64.1 (22.2-105.9)	
2005	All	43	16.2 (11.4-21.0)	18.8 (13.2-24.5)
	<45	8	9.0 (2.8-15.3)	
	45–54	9	35.6 (12.3-58.8)	
	55–64	11	74.7 (30.6-118.9)	
	65+	15	106.9 (52.6-160.5)	
2006	All	34	12.6 (8.4-16.9)	14.7 (9.8-19.6)
	<45	9	10.0 (3.5-16.6)	
	45–54	6	24.4 (4.9-44.0)	
	55–64	9	63.9 (22.2-105.7)	
	65+	10	79.5 (30.2-128.8)	
	Unknown			

**Figure 1:**
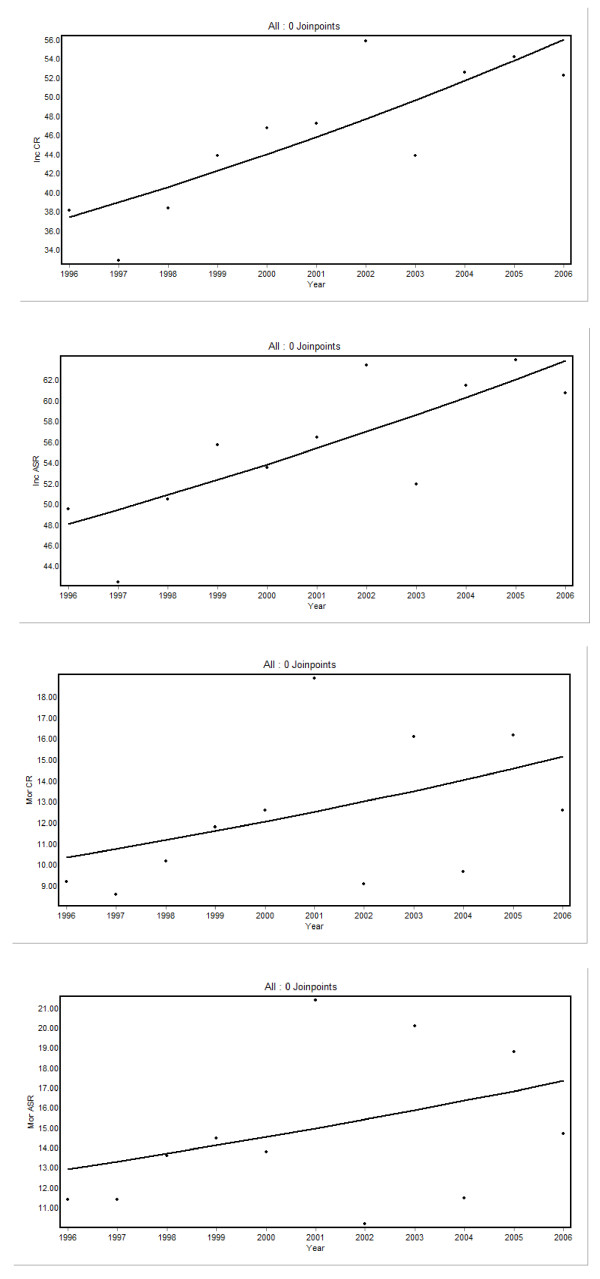
Crude (Inc CR; 95% CI: 2.0 to 6.3) and age-standardized (Inc ASR; 95% CI: 1.1 to 4.6) breast cancer incidence rates and crude (Mor CR; 95% CI: -2.2 to 10.3) and age-standardized (Mor ASR; 95% CI: -2.8 to 9.1) mortality rates, Aracaju, Sergipe, Brazil, 1996–2006).

As seen in Table 3, trends in breast cancer incidence and mortality were calculated using Joinpoint analyses for age-standardized, crude and age-specific rates. Across all age groups, there was an increasing trend, with an ASR-APC of 2.9 (95% CI: 1.1 to 4.6) and a CR-APC of 4.1 (95% CI: 2.0 to 6.3). In the groups aged 45–54 and 55–64 years, the APC was 3.9 (95% CI: 1.4 to 6.6) and 5.6 (95% CI: 1.8 to 9.6), respectively. For mortality, the overall trend was not statistically significant, with an ASR-APC of 3.0 (95% CI: -2.8 to 9.1) and a CR-APC of 3.9 (95% CI: -2.2 to 10.3). The only significant mortality trend was observed in the group aged 55–64 years, with an APC of 11.3 (95% CI: 1.1 to 22.4). The incidence and mortality trend curves are displayed in Figure 1.

**Table 3:** Joinpoint analyses of breast cancer incidence and mortality with the estimated annual percent change (APC) for age-specific groups, crude rates (CR), age-standardized rates (ASR), and 95% confidence intervals (CI), Aracaju, Sergipe, Brazil, 1996–2006

	**Incidence**		**Mortality**	
**Age groups**	**Join points**	**APC (95% CI)**	**Join points**	**APC (95% CI)**
All (ASR)	0	2.9* (1.1-4.6)	0	3.0 (−2,8-9.1)
All (CR)	0	4.1* (2.0-6.3)	0	3.9 (−2.2-10.3)
<45	0	1.8 (−1.9-5.6)	0	0.8 (−5.0-6.9)
45-54	0	3.9* (1.4-6.6)	0	−3.8 (−16.2-10.5)
55-64	0	5.6* (1.8-9.6)	0	11.3* (1.1-22.4)
65+	0	4.1 (−0.5-8.9)	0	−0.4 (−7.2-6.9)

*Significant APC.

## Discussion

Breast cancer incidence is higher in high-income countries, because of its links with several risk factors [16] and the presence of systematic screening policies [17]. However, a decreasing trend has been observed since 2003 due to the decreasing use of HRT [18]. Lower incidence rates in low-income countries probably reflect international variation in hormonal factors and accessibility to early detection facilities [19,20].

Screening practices in Brazil were outlined by the Brazilian National Cancer Institute [21] and have been followed by the states and municipalities. Screening guidelines include biannual mammography for women aged 50 to 69 years, and have been made public through occasional media campaigns.

In the study period, increasing CRs and ASRs were observed (Table 1 and Figure 1). In 1996, the ASR was 49.6/100,000 (95% CI: 39.2 to 60.0). In the following years, it had some fluctuation before becoming stable with rates higher than 60.0/100,000 in the last years of the series. Rates as high as these have been observed in other mid-income countries [22] and could be justified in an area with an intermediate HDI such as Aracaju.

Increasing trends (Table 3) were observed in the study, with an ASR-APC of 2.9 (95% CI: 1.1 to 4.6) and a CR-APC of 4.1 (95% CI: 2.0 to 6.3). Incidence rates were higher in peri- and postmenopausal women, with increasing trends in the groups aged 45–54 (APC: 3.9, 95% CI: 1.4 to 6.6) and 55–64 (APC: 5.6, 95% CI: 1.8 to 9.6) years. In the study community, formal screening invitations are not conducted. Rather, mammography is offered to women when they present to their health care practitioner because of other health problems, or even to request screening. This might somehow reflect the increasing trends in these age groups. Screening has been more consistently available since the early 1990s, when breast specialists began practicing in this community. In addition, first generation high-resolution breast imaging has also become available. Screening guidelines were established in 2004 on a biannual basis for women aged 50 to 69 years. The number of women in different age groups actually being screened is unknown. Furthermore, the role of HRT could not be established and might be worthy of future research.

Mortality rates are higher in high-income countries, but with decreasing trends, with a median ASR of 15.3/100,000 [22,23]. In the United States, for instance, mortality rates standardized by the American population varied from 21.5/100,000 to 28.0/100,000, depending on the state, but have also presented decreasing trends [24]. Low-income areas have shown a median ASR of 10.0/100,000, but with increasing trends [22,23]. Possible explanations for this are the low incidence rates observed in those areas, and the lack of risk factors typically seen in high-income countries. Regarding increasing trends, inefficacy to detect less advanced tumors and lower accessibility to treatment facilities were suggested [19,20]. In our study, crude and age-standardized mortality rates fluctuated throughout the years. A mean age-standardized mortality rate of 14.7/100,000 was observed in the 1996–2006 period. No significant trends were observed, with an ASR-APC of 3.0/100,000 (95% CI: -2.8 to 9.1) and CR-APC of 3.9 (95% CI: -2.2 to 10.3). However, there has been an increasing trend for the group aged 55–64 years, with an APC of 11.3 (95% CI: 1.1 to 22.4), probably reflecting the trend of increasing incidence in the peri- and postmenopausal periods. Actual cause of death has been a matter of discussion, because it has not always been adequately interpreted. In our study, breast cancer death was considered when it was certified as the underlying or contributing cause.

The mortality-to-incidence ratio has been advocated as a proxy for cancer survival, taken by 1-(M/R) [25]. Mortality-to-incidence ratios around 0.24 have been observed in high-income countries, compared with 0.40 in less developed and 0.60 in low-income areas such as Africa [26]. In the present study, a mean ratio of 0.27, which is similar to those of high-income areas, is rather satisfactory, but might be confirmed more accurately by survival studies.

It has been suggested that systematic screening, together with implementing better treatment facilities is the key to decreasing mortality rates [27,28], but screening is sometimes contested because of the high cost of establishing mammography equipment, the low yield of biopsies, the lead time bias, and the diagnosis of lesions that would never become invasive [17].

### Limitations

The Population-Based Cancer Registry of Aracaju has registered incident cancer cases in the State of Sergipe and has then selected cases from the area of Aracaju. This has resulted in a delay to close the annual database and case ascertainment has been more tedious. There have been some cases for whom place of residence could not be determined. Even after examining all sources and databases, a few cases still had to be excluded. The data were retrieved from several different sources of information, and some cases could be found in more than one information source; therefore, extra care had to be exerted to avoid duplication. Mortality rates were calculated from death records of the Official State Mortality Database. The cause of death has been criticized as being inadequately precise, mainly in developing areas, which jeopardizes our conclusions. However, official data were used and all possible effort was put into improving the quality of information.

## Conclusions

The present study demonstrated that breast cancer incidence has been increasing over time. This increasing trend may continue more steadily with the aging of the still-young female population and if more systematic screening policies are implemented. Mortality trends were not observed to be increasing, except for the group aged 55–64 years. Control policies should aim to more accurately screen women in the peri- and postmenopausal periods to diagnose smaller tumors that could have better outcomes. It was not the aim of this study to analyze the causes of the increase in incidence and the impact on mortality rates. Additional research needs to be conducted to identify factors that could be related to this crescent trend to better design strategies to minimize the impact on mortality.

N, Number of cases; se, standard error.

## Abbreviations

CI: Confidence interval; HRT: Hormone replacement therapy; HDI: Human development index; IARC: International agency for research on cancer; INCA: Instituto Nacional de Câncer; CRs: Crude rates; ASRs: Age-standardized rates; APC: Annual percent change; M/I: Mortality to incidence ratio; R: Rate.

## Competing interests

The authors declare that they have no conflict of interest.

## Authors’ contributions

CAL and AMS designed the study, had access to the database and performed data analysis. Additional analysis and interpretation were undertaken by CAL, AMS, and MRR. CAL drafted the manuscript. Revision of the manuscript was conducted by MML and AMS. All authors read and approved the final manuscript.

## Pre-publication history

The pre-publication history for this paper can be accessed here:

http://www.biomedcentral.com/1471-2458/12/883/prepub
